# Evidence of Spatially Extensive Resistance to PCBs in an Anadromous Fish of the Hudson River

**DOI:** 10.1289/ehp.8255

**Published:** 2005-09-21

**Authors:** Zhanpeng Yuan, Simon Courtenay, R. Christopher Chambers, Isaac Wirgin

**Affiliations:** 1 Department of Environmental Medicine, New York University School of Medicine, Tuxedo, New York, USA; 2 Department of Fisheries and Oceans, Gulf Region, Canadian Rivers Institute/Department of Biology, University of New Brunswick, Fredericton, New Brunswick, Canada; 3 Howard Marine Sciences Laboratory, Northeast Fisheries Science Center, National Oceanic and Atmospheric Administration Fisheries Service, Highlands, New Jersey, USA

**Keywords:** AHR, Atlantic tomcod, CYP1A, evolutionary change, genetic adaptation, Hudson River, PCBs, resistance

## Abstract

Populations of organisms that are chronically exposed to high levels of chemical contaminants may not suffer the same sublethal or lethal effects as naive populations, a phenomenon called resistance. Atlantic tomcod (*Microgadus tomcod*) from the Hudson River, New York, are exposed to high concentrations of polycyclic aromatic hydrocarbons (PAHs) and bioaccumulate polychlorinated biphenyls (PCBs), polychlorinated dibenzo-*p*-dioxins (PCDDs), and polychlorinated dibenzofurans (PCDFs). They have developed resistance to PCBs and PCDDs but not to PAHs. Resistance is largely heritable and manifests at early-life-stage toxic end points and in inducibility of cytochrome P4501A (CYP1A) mRNA expression. Because *CYP1A* induction is activated by the aryl hydrocarbon receptor (AHR) pathway, as are most toxic responses to these compounds, we sought to determine the geographic extent of resistance to CYP1A mRNA induction by PCBs in the Hudson River tomcod population. Samples of young-of-the-year tomcod were collected from seven locales in the Hudson River, extending from the Battery at river mile 1 (RM 1) to RM 90, and from the Miramichi River, New Brunswick, Canada. Laboratory-reared offspring of tomcod adults from Newark Bay, in the western portion of the Hudson River estuary, were also used in this study. Fish were partially depurated in clean water and intraperitoneally injected with 10 ppm coplanar PCB-77, 10 ppm benzo[*a*]pyrene (BaP), or corn oil vehicle, and levels of CYP1A mRNA were determined. *CYP1A* was significantly inducible by treatment with BaP in tomcod from the Miramichi River, from laboratory-spawned offspring of Newark Bay origin, and from all Hudson River sites spanning 90 miles of river. In contrast, only tomcod from the Miramichi River displayed significantly induced CYP1A mRNA expression when treated with PCB-77. Our results suggest that the population of tomcod from throughout the Hudson River estuary has developed resistance to *CYP1A* inducibility and probably other toxicities mediated by the AHR pathway. Tomcod from the Hudson River may represent the most geographically expansive population of vertebrates with resistance to chemical pollutants that has been characterized.

Resistance to toxicants has been reported in populations of invertebrates (Klerks and Levinton 1989) and fish (reviewed by Wirgin and Waldman 2004) from highly polluted aquatic ecosystems. For example, populations of Atlantic killifish, *Fundulus heteroclitus*, from three highly contaminated estuaries along the Atlantic Coast of the United States exhibit dramatic resistance to aromatic hydrocarbon (AH) compounds, including polychlorinated biphenyls (PCBs) (Bello 1999; Elskus et al. 1999; Nacci et al. 1999), 2,3,7,8-tetra-chlorodibenzo-*p*-dioxin (TCDD) (Prince and Cooper 1995a, 1995b), and creosote-containing polycyclic aromatic hydrocarbons (PAHs) (Meyer et al. 2002; Van Veld and Westbrook 1995). Resistance to AH contaminants in these populations has been observed at the molecular, biochemical, and organism levels, as evidenced by significantly reduced inducibility of cytochrome P4501A (*CYP1A*) and reduced sensitivities of early life stages to toxicities elicited by exposures to these chemicals. In these cases of resistance, sensitivities to chemicals are usually not completely abolished but are reduced by one or two orders of magnitude relative to responsive populations.

Resistance can result from genetic adaptations from chronic exposures of populations to contaminants, in which case resistance will usually persist for many generations after remediation of the environment. If an ecosystem harbors a single panmictic population of the affected species, the entire population may develop resistance. If individuals persist in a fragmented mosaic of populations with limited gene flow among subgroups, then some subpopulations may show little or at least varying degrees of resistance to the toxicant. Alternatively, resistance can result from physiologic acclimation(s) without an underlying genetic basis, in which case resistance will be quickly lost after cessation of exposures to contaminants. Physiologic acclimation should be expressed only by highly exposed individuals and may not be detected in all members of even the local subpopulation. To date, studies on killifish indicate that resistance is largely a genetically based phenotype (Bello et al. 2001; Meyer and Di Giulio 2002, 2003; Nacci et al. 1999), although evidence exists in one affected population of a nongenetic, physiologic role in the resistance phenotypes (Meyer et al. 2002). Studies that have attempted to elucidate the mechanistic basis of resistance in these populations have focused on components of the aryl hydrocarbon receptor (AHR) pathway (reviewed by Wirgin and Waldman 2004), which activates transcription of CYP1A mRNA and mediates most overt toxicities to AH compounds in mammals (Ma 2001) and fishes (Hahn 1998).

Irrespective of its mechanistic basis, resistance of natural populations to toxicants is believed to involve trade-offs associated with constraints imposed by a limited energy budget. Evidence of such trade-offs includes increased sensitivities to other stressors or reduced performance in the absence of contaminants (McKenzie 1996; Shaw 1999). Effects of resistance may also be evident at the community level. Contaminants may be transferred up the food chain because resistant populations survive to be consumed by predators. If contaminant transfer occurs, it is likely to result in ever-greater levels of contaminants at higher trophic levels if the resistant population is prey consumed by an array of consumers. The abundance and trophic importance of a resistant population are therefore likely to be key factors in determining the magnitude of bioaccumulation at higher trophic levels. Furthermore, for chemicals such as polychlorinated dibenzo-*p*-dioxins (PCDDs), polychlorinated dibenzofurans (PCDFs), and PCBs, which are highly refractory to metabolism in fishes, trophic transfer of contaminants by resistant populations may be particularly profound. As a result, the geographic extent of resistance in a population is important in predicting its consequences at the population, community, and ecosystem levels.

Most evidence of resistance in aquatic ecosystems has been from populations with limited geographic distributions. The spatial restriction of resistant populations studied to date is likely a function of the distribution of the contaminants that historically elicited a toxic response and the relatively low mobility of affected populations. For example, a resistant population of the oligochaete worm *Limnodrilus hoffmeisteri* was restricted to one cove of the Hudson River where unusually high levels of metals were detected in the sediments (Klerks and Levinton 1989). Similarly, resistance in populations of killifish in New Bedford Harbor, Massachusetts (Nacci et al. 2002a) and the Elizabeth River, Virginia (Ownby et al. 2002) was limited to within several kilometers of sites known for high concentrations of PCBs and PAHs, respectively, at those locales. In these examples, the limited spatial distribution of resistance is consistent with the limited mobility (Lotrich 1975) of the focal species and, hence, negligible levels of gene flow among populations (Mulvey et al. 2002, 2003).

## Contamination of the Hudson River estuary.

More than 200 miles of the Hudson River is a U.S. federal Superfund site because of the release of PCBs for more than four decades from two manufacturing facilities at river mile (RM) 195 and RM 197 (Farley and Thomann 1998). PCBs from these upriver sources were transported downriver and resulted in an inverse concentration gradient between levels of contaminants in Hudson River sediments, as well as tissue burdens in fishes, and distance downstream. Major secondary peaks of PCBs in sediments and fishes have been observed in the vicinity of New York City and are believed to originate at local municipal and industrial sources. The Passaic River, one of two tributaries of Newark Bay in the western portion of the lower Hudson River estuary, is also designated as a federal Superfund site because of high levels of contamination with TCDD that originated from the Diamond Alkali herbicide manufacturing facility situated on its banks. TCDD has been transported downstream and has contaminated much of Newark Bay (Bopp et al. 1991). Finally, levels of PAHs in the sediments of the lower Hudson River estuary were the second highest of any U.S. estuary surveyed in the mid-1980s [National Oceanic and Atmospheric Administration (NOAA) 1988].

## Life history characteristics of Atlantic tomcod.

Atlantic tomcod, *Microgadus tomcod*, is a common anadromous species in many estuaries along the Atlantic Coast of North America from the Hudson River to Labrador, Canada (Bigelow and Schroeder 1953). Within the Hudson River estuary, juvenile and adult tomcod are seasonally distributed over 145 miles of Hudson River from the Battery at RM 1 to Albany at RM 145 (Klauda et al. 1988) and in Newark Bay and its tributaries. Estimated population sizes for adult tomcod in the Hudson River range from 0.8 to 3.0 × 10^6^ fish (Draft Environmental Impact Statement 1999), with a generation time of approximately 1.1 years (Mattson M, personal communication).

Throughout its range, tomcod spawn in midwinter. The winter spawning of tomcod is unique among the ichthyofauna of the lower Hudson River estuary. In addition to a protracted benthic embryonic period due to annual minimum water temperatures experienced by embryos, the timing of spawning results in young life stages of tomcod being a key prey item for major resource species and other ecologically important species within the lower estuary in spring and early summer (Dew and Hecht 1994). Key consumers of tomcod, among others, include bluefish (*Pomatomus saltatrix*), striped bass (*Morone saxatilis*), weakfish (*Cynoscion regalis*), summer flounder (*Paralichthys dentatus*), American eel (*Anguilla rostrata*), and white catfish (*Ictalurus catus*). Examination of the gut content of these consumer species indicates that tomcod is their dominant prey when these species are associated with the bottom habitat of the Hudson River during the spring and summer months (Chambers and Witting, in press).

The entire life cycle of tomcod occurs within or in close proximity to their natal estuaries. Adults are highly migratory within the confines of their natal estuaries, with a dominant seasonal relocation in autumn to upstream locations for spawning (Klauda et al. 1988). Because of the mobility of adults, and because of their more diverse diets—including other fishes—the exposure experience of adult tomcod is likely to be more a reflection of estuary-wide contaminant levels than would be true for younger tomcod life stages. Tomcod are associated with the river bottom in all life stages except larval life and thus may be exposed to lipophilic AHs via direct contact with sediments, ingestion of sediments, and consumption of benthic prey. Juvenile and adult tomcod from the Hudson River have unusually high levels of hepatic lipids that may further serve to bioaccumulate unusually high levels of lipophilic contaminants (Cormier et al. 1989).

## Contamination of tomcod from the Hudson River.

PAHs cannot be directly measured in fish livers because of their rapid metabolism (Krahn et al. 1986). Surrogate measures of PAH exposure have been developed, and these revealed high levels of PAH metabolites in bile and hepatic DNA adducts (also a signature of PAH exposure) in adult tomcod from the Hudson River (Wirgin et al. 1994). Furthermore, elevated levels of persistent hepatic PCBs and PCDDs/PCDFs were detected in adult tomcod from the Hudson River (Courtenay et al. 1999), in their unfertilized eggs (Roy et al. 2001), and in juvenile tomcod (Yuan et al. 2001). Further studies quantified congener-specific levels of hepatic PCBs and PCDDs/PCDFs in adult and juvenile tomcod at 20 locations in the Hudson River estuary extending from the Battery at RM 0 to RM 107, in the Hackensack River (the second of two tributaries of Newark Bay), and in Newark Bay. These studies showed major differences among collection sites in liver burdens and homologue/congener composition of PCBs (Fernandez et al. 2004).

## Perturbations in tomcod from the Hudson River.

Tomcod from the Hudson River exhibited one of the highest prevalences of tumors ever observed in a natural population. In the early 1980s, ≥ 40% of 1-year-old and 90% of 2-year-old Hudson River tomcod exhibited hepatocellular carcinomas (Dey et al. 1993). Tumors were absent (Couillard et al. 1999) or had < 5% prevalences in tomcod from cleaner rivers (Cormier and Racine 1990). Concurrently, the tomcod population in the Hudson River exhibited a truncated age-class structure compared with those from elsewhere. However, the prevalence of gross hepatic lesions has decreased (Young J, personal communication), and the longevity in the population has increased in recent years (New York State Department of Environmental Conservation 2003).

## CYP1A *function and its use as a bio-marker in tomcod.*

Levels of CYP1A mRNA, protein, and encoded enzyme are widely used in fishes as biomarkers of exposure to AHs and their early biologic effect (Wirgin and Theodorakis 2002). *CYP1A* expression in fishes is inducible by exposure to PCDDs/PCDFs, coplanar PCBs, and some PAHs (Stegeman and Hahn 1994) and is usually a sensitive, dose-responsive, reversible, and specific biomarker to these contaminants and may be predictive of some higher level toxic effects. Usually, PCDDs/PCDFs are the most potent inducers of *CYP1A*, and PAHs the least. Levels of *CYP1A* expression in fish populations have been correlated with higher level biologic effects such as DNA damage (Wirgin et al. 1994), prevalence of hepatic neoplasms (Stein et al. 1994), teratogenicity, and increased early-life-stage mortality (Wright and Tillitt 1999).

Environmentally exposed adult tomcod collected from the Hudson River and immediately sacrificed exhibited significantly higher expression of hepatic CYP1A mRNA expression than did tomcod from four cleaner rivers (Kreamer et al. 1991; Wirgin et al. 1994). In controlled laboratory studies, however, CYP1A mRNA was not inducible in tomcod from the Hudson River that were extensively depurated and then treated with PCB-77 (0.1–10 mg/kg fish) or TCDD (100 ng/kg fish) (Wirgin et al. 1992). In contrast, *CYP1A* was highly inducible in Hudson River tomcod treated with benzo[*a*]pyrene (BaP) or β-naphthoflavone (β-NF). Furthermore, *CYP1A* was highly inducible with PCB-77, TCDD, BaP, and β-NF in tomcod from the cleaner Miramichi River (New Brunswick, Canada). In later, more intensive studies, significant CYP1A mRNA inducibility occurred in tomcod from the Hudson River at concentrations of halogenated aromatic hydrocarbons (HAHs) two orders of magnitude higher than in tomcod from relatively pristine sites. Furthermore, the reduced sensitivity of Hudson River tomcod occurred in all tissues and at all life stages (Yuan et al. 2005). Additionally, studies showed that reduced inducibility of CYP1A mRNA by PCB-77 was in part heritable to the F_1_ larvae (Roy et al. 2001) and more so to F_2_ embryos (Wirgin II, unpublished data).

In this study, we sought to determine the geographic distribution of resistance in the Hudson River population of tomcod. Because almost 200 miles of the Hudson River are highly contaminated with PCBs and in the absence of any information of the reproductive fragmentation of the Hudson River population, we hypothesized that tomcod from throughout its 145-mile distribution in the Hudson River would exhibit significantly reduced inducibility of *CYP1A* compared with those from cleaner locales. From a management perspective, this information is important in assessing the potential risks of contamination of tomcod to the Hudson River ecosystem.

## Materials and Methods

### Source of juvenile tomcod.

*CYP1A* inducibility and constitutive expression in fishes vary with life stage, sex, and reproductive status with respect to the spawning season (Elskus et al. 1992). Less variation occurs in *CYP1A* inducibility among juvenile fish than for adults, including tomcod (Williams et al. 1998). Because the spatial distribution of juveniles is more localized than that of adults, CYP1A mRNA expression in juveniles is more likely to reflect the fine-scale distribution of bioavailable contaminants.

**Figure 1:**
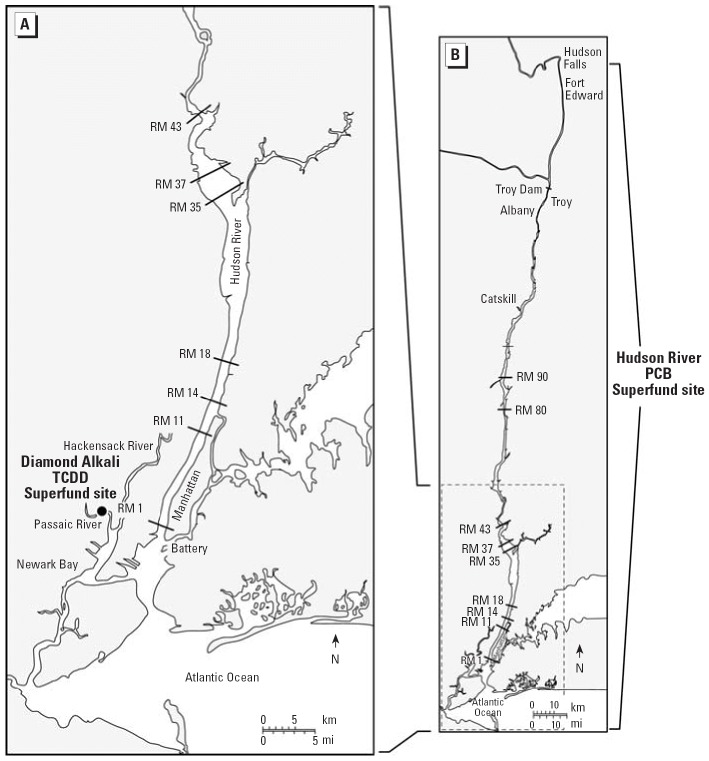
Map of the Hudson River estuary indicating sites from which tomcod were collected for this study and others cited in the text. (*A*) is an enlargement of the boxed area in (*B*).

Because tomcod in the Hudson River mature at 1 year of age, all juveniles are young-of-the year fish. We used three sources for juvenile tomcod in this study. One source was the Hudson River, where tomcod were collected by trawling during the summer months of 2000 and 2003. Fish were collected from seven sites in the Hudson River estuary (RM 1, RM 11, RM 18, RM 37, RM 43, RM 80, and RM 90) (Figure 1) and transported to the laboratory, where they were maintained separately by site in 100-gallon aquaria filled with 20 ppt seawater at 12°C for at least 21 days before experimentation. A second source of juvenile tomcod was the Miramichi River, where tomcod were collected with beach seines in June 2004. Hepatic levels of PCBs and PCDDs/PCDFs in juvenile tomcod from the collection sites or nearby sites for both the Hudson River and the Miramichi River are presented in Table 1. For the third source of juvenile tomcod, we used offspring of wild-caught adults collected from Newark Bay, New Jersey, by otter trawl in December 2000. Total TCDD toxic equivalent quotients (TEQs) in adult tomcod collected in 1998 from Newark Bay are also presented in Table 1. The adults were sent to the Howard Marine Sciences Laboratory (NOAA Fisheries Service, Highlands, NJ, USA) where, when ripened, they were stripped of gametes to be used for fertilization. Embryos and larvae were grown under controlled conditions in the laboratory, and juveniles were maintained until use in this study.

### Experimental design.

All fish were weighed before experimentation, and then each source of fish was treated in one of three ways. One group from each source was injected intraperitoneally (ip) with 10 ppm PCB-77. A second group received an ip injection of 10 ppm BaP. A third group received an ip injection of corn oil vehicle. Treated fish were maintained in the laboratory and then sacrificed after either 7 days (PCB-77), 2 days (BaP), or both 2 and 7 days (corn oil). In previous extensive kinetic experiments, we determined that maximum levels of induction of CYP1A mRNA in tomcod occurred at these times after treatment with these chemicals (Courtenay et al. 1999). PCB-77 is very persistent in tomcod livers, whereas PAHs such as BaP are rapidly metabolized and cleared. Livers of treated fish were dissected and frozen at –80°C until processed.

### Preparation of RNA samples.

Total RNA was isolated from approximately 50 mg of liver tissue per specimen. Tissues were homogenized in RNAzol or Ultraspec reagent (both from BIOTECX Laboratories Inc., Houston, TX, USA) in 1.5 mL microcentrifuge tubes, and RNAs were isolated as recommended by the manufacturer and modified as described by Courtenay et al. (1999). RNA pellets were washed twice in 75% ethanol, resuspended in 50 μL of diethylpyrocarbonate (DEPC)-treated water, and stored at –80°C until further processing. Concentrations and purities of RNA samples were determined by ultraviolet (UV) spectrophotometry at 260 and 280 nm.

All RNA samples were further analyzed for integrity using denaturing Northern gels. Two micrograms of each RNA was denatured and loaded into 1% agarose gels cast in 1× MOPS buffer containing 1% formaldehyde (vol/vol), stained in ethidium bromide solution, and photographed under UV illumination to evaluate the integrity of the 28S and 18S ribosomal RNA bands (Courtenay et al. 1999). Degraded RNAs were discarded and RNAs from these tissues were re-isolated.

### CYP1A mRNA quantification by slot blot hybridization.

Two micrograms of RNA samples were denatured and applied onto Nytran Nylon Plus membranes (Schleicher & Schuell, Keene, NH, USA) using a 72-well slot blot apparatus (Schleicher & Schuell) as described by Courtenay et al. (1999).

Full-length *CYP1A* cDNA isolated from a β-NF–treated Hudson River tomcod (Roy et al. 1995) (GenBank accession no. L41886; Genbank 1996) and full-length rat 18S rRNA cDNA (Chan et al. 1984) were labeled with ^32^P using Random Priming Kits (Roche Diagnostics Corp., Indianapolis, IN, USA) according to the the manufacturer’s instructions; we used these to probe the blots.

Membranes were prepared, prehybridized, and hybridized overnight at 65°C as described by Courtenay et al. (1999). After hybridizations, the membranes were washed three times in 6× saline-sodium phosphate-EDTA (SSPE)/0.1% at room temperature for 5 min each and twice in 1× SSPE/0.1% sodium dodecyl sulfate (SDS) at 65°C for a total of 1 hr. CYP1A mRNA levels were quantified from phosphor imaging screens using a Storm 860 scanner and ImageQuant for Macintosh software (version 1.0; Molecular Dynamics, Sunnyvale, CA, USA). *CYP1A* probes were then stripped off membranes by twice immersing them in boiling 0.1× standard sodium citrate/0.5% SDS while shaking. The membranes were then prehybridized and hybridized with 18S rRNA probes and quantified as above.

The length of time for which membranes were phosphor imaged for both *CYP1A* and 18S rRNA varied and therefore resulted in differing absolute optical density (OD) units among blots.

### Statistical analysis.

We conducted three sets of statistical analyses. All used CYP1A mRNA concentrations (relative OD units) as proxies for a toxic response. First, we evaluated juvenile tomcod from three Hudson River sites (RM 11, RM 18, and RM 37) for spatial differences in CYP1A mRNA concentrations. Second, we compared juvenile Hudson River tomcod with juvenile tomcod from the Miramichi River. Third, juvenile tomcod from four Hudson River sites (RM 1, RM 43, RM 80, and RM 90) were compared with laboratory-reared tomcod juveniles from Newark Bay in the western Hudson River estuary.

For the second analyses, the CYP1A mRNA levels of the Miramichi River juveniles injected with corn oil vehicle did not differ between times of sacrifice (i.e., 2 vs. 7 days), so these two groups were pooled for comparison with other treatments. Similarly, Hudson River tomcod from the three sites (RM 11, RM 18, RM 37) did not differ in their responses to each of the three ip treatments, so data from the three sites were pooled before comparison with responses from vehicle controls and from Miramichi River tomcod.

In all cases, CYP1A mRNA data were normalized to respective 18S rRNA concentrations, and the results were log-transformed to improve distribution normality. *CYP1A* data were compared by analysis of variance (ANOVA) followed by the Tukey multiple range test. Means and 95% confidence intervals (CIs) were back-transformed to original units for presentation.

## Results

### *Site differences in Hudson River tomcod* CYP1A *levels.*

**Figure 2:**
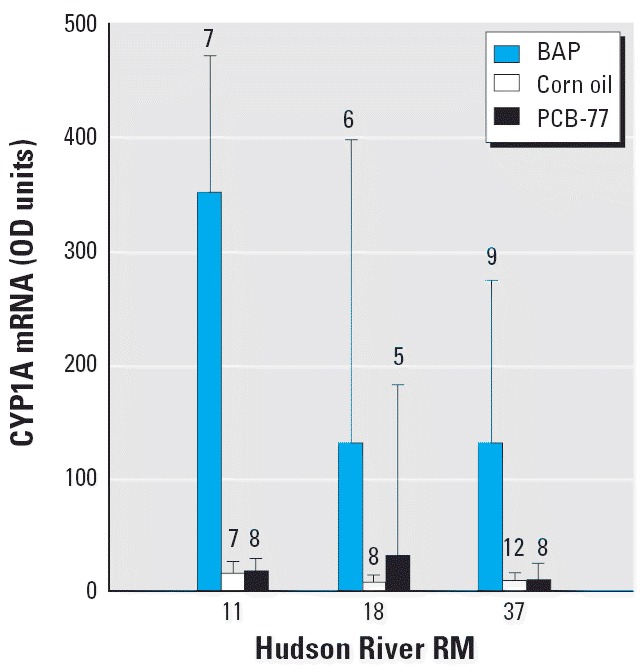
CYP1A mRNA expression levels (mean and 95% CIs, expressed in OD units) in juvenile tomcod sampled from three sites in the Hudson River, depurated in the laboratory for 21 days, and then injected ip with 10 ppm BaP, corn oil vehicle, or 10 ppm PCB-77. Data are back-transformed from log-transformed data used for ANOVA comparisons. Numbers above bars represent sample size.

Juvenile tomcod from three Hudson River sites, RM 11, RM 18, and RM 37, that were depurated and injected ip with 10 ppm of BaP, a potent PAH-type inducer of *CYP1A*, did not differ from one another but did exhibit significantly higher CYP1A mRNA concentrations than did vehicle-treated controls collected from the same sites (one-way ANOVA *F*_2,67_ = 55.288, *p* < 0.001; Tukey test *p* < 0.001) (Figure 2). By contrast, juvenile Hudson River tomcod from all three sites that were injected ip with 10 ppm PCB-77 showed no induction above controls. CYP1A mRNA concentrations were significantly higher in chemically treated tomcod from RM 11 than in those from RM 37 (two-way ANOVA *F*_2,61_ = 4.447, *p* = 0.016; Tukey test *p* = 0.011) for both BaP and PCB-77 treatments (nonsignificant interaction term in two-way ANOVA).

### *Differences in* CYP1A *levels between Hudson River and Miramichi River tomcod.*

**Figure 3:**
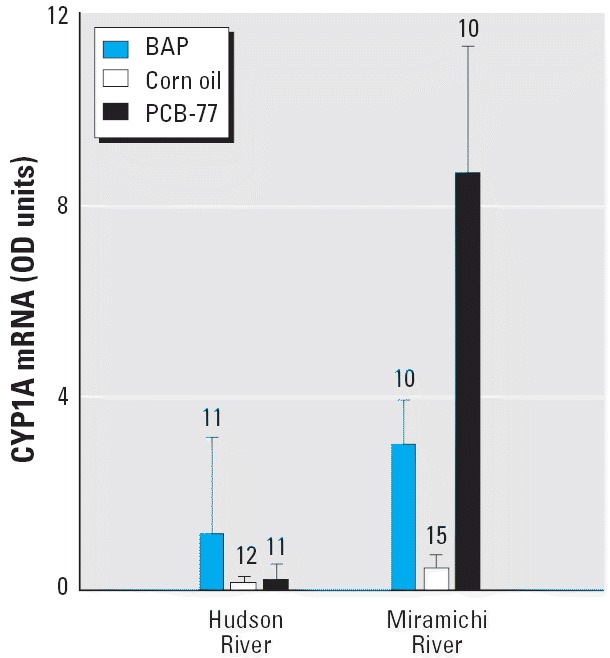
CYP1A mRNA expression levels (mean and 95% CIs, expressed in OD units) in juvenile tomcod from the Hudson River (HR) and Miramichi River (MR) injected ip with 10 ppm BaP, corn oil vehicle, or 10 ppm PCB-77. Data are back-transformed from log-transformed data used for ANOVA comparisons. Numbers above bars represent sample size.

A subset of Hudson River juveniles from the three sites (RM 11, RM 18, and RM 37; three to five fish per treatment per site) were compared with Miramichi River tomcod for CYP1A mRNA response to ip injection with 10 ppm BaP and 10 ppm PCB-77 (Figure 3). Fish from the two populations responded differently to one or both chemical treatments (two-way ANOVA, population × treatment interaction: *F*_2,63_ = 9.081, *p* < 0.001). Separate comparison of each chemical with the control indicated that the population difference in response was to PCB-77 (interaction term: F_1,44_ = 11.249, *p* = 0.002). Consistent with the previous results, the Hudson River tomcod were relatively insensitive to PCB-77 compared with the response elicited in Miramichi River tomcod. Both populations responded similarly to BaP (interaction term: F_1,44_ = 0.689, *p* = 0.411).

### *Differences in* CYP1A *levels between wild and laboratory-reared Hudson River tomcod.*

**Figure 4:**
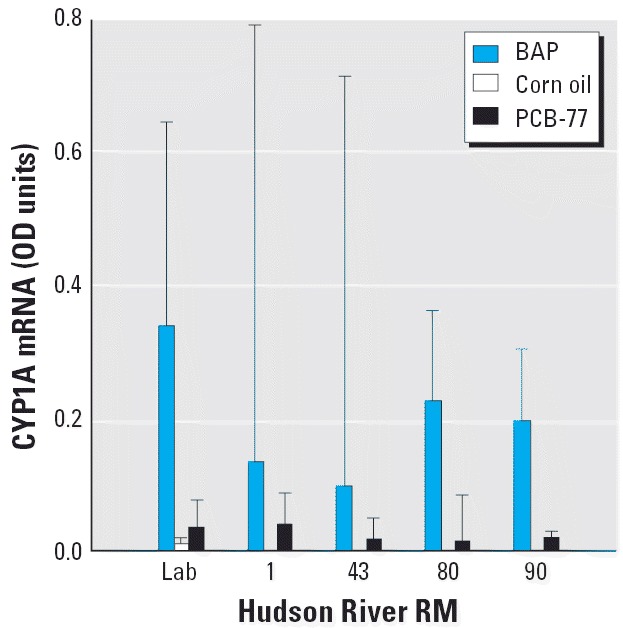
CYP1A mRNA expression levels (mean and 95% CIs, expressed in OD units) in juvenile Hudson River tomcod injected ip with 10 ppm BaP, corn oil vehicle (in Lab group only), or 10 ppm PCB-77. Lab indicates Newark Bay tomcod spawned in the laboratory; other fish were sampled from different RMs of the Hudson River. Data are back-transformed from log-transformed data used for ANOVA comparisons; *n* = 4–5/group, except for Lab control, where *n* = 11.

Juvenile tomcod sampled from various Hudson River locations (RM 1, RM 43, RM 80, and RM 90) and laboratory-reared offspring of approximately the same age from the western Hudson River estuary all responded significantly and similarly to ip injection of 10 ppm BaP when compared with laboratory-reared corn-oil control fish (one-way ANOVA *F*_5,28_ = 11.694, *p* < 0.001; Tukey test, *p* ≤ 0.006 for all groups compared with controls) (Figure 4). Furthermore, there were no significant differences among BaP-injected groups in magnitude of induction (*p* > 0.4, Tukey test). By contrast, significant induction over controls was not observed in any of the field-sampled or laboratory-reared tomcod injected ip with PCB-77 (*p* > 0.5, Tukey test). CYP1A mRNA concentrations were similarly low among all PCB-77–injected groups (*p* > 0.5, Tukey test).

## Discussion

Juvenile Atlantic tomcod from a wide range of locations (90 miles) in the Hudson River appear to be resistant to induction of CYP1A mRNA by coplanar PCB congener 77. Additionally, resistance was observed in the laboratory-reared offspring of parents collected in Newark Bay in the western estuary. In contrast, CYP1A mRNA was significantly inducible by treatment with BaP in tomcod from all Hudson River sites and in laboratory-reared fish. The distribution of juvenile tomcod in the Hudson River in some years extends from the river’s mouth to Albany, New York, at RM 145 and includes the western estuary. Thus, juvenile tomcod from a large proportion of its range in the Hudson River estuary exhibit resistance to *CYP1A* inducibility and perhaps other toxicities mediated by the AHR pathway. There was no significant difference among collection sites in *CYP1A* expression levels of fish treated with PCB-77, suggesting that the degree of resistance was the same in fish collected from all Hudson River locales. In contrast, juvenile tomcod from the Miramichi River exhibited significant induction of CYP1A mRNA after treatment with either BaP or PCB-77.

Almost 200 miles of Hudson River is a federal Superfund site because of PCB contamination of sediments and biota (Wirgin et al. 2005). PAHs also contaminate much of the lower estuary at levels that are among the highest of any U.S. estuary (NOAA 1988). The Passaic River is also a Superfund site because of TCDD contamination, and much of Newark Bay and the Hackensack River are highly polluted with TCDD, PCBs, and PAHs. Tomcod from throughout the estuary are exposed to and bioaccumulate these contaminants, and the resistance of this population is consistent with an estuary-wide exposure to elevated levels of these contaminants. There is considerable variation, however, in tissue burdens of HAH contaminants in juvenile tomcod collected from multiple locales within the estuary. For example, significant spatial variation in PCB levels and congener patterns have been observed among juvenile tomcod. Three statistical clusters were identified in juveniles collected from RM 1 to RM 17, from RM 37 to RM 77, and from RM 80 to RM 107 (Fernandez et al. 2004). Despite their differing exposure histories, groups of juvenile tomcod from all three clusters exhibit resistance to PCB treatment.

Resistance of tomcod to HAHs over such a large portion of its distribution is consistent with its life history and exposure history in the Hudson River estuary. There are no known barriers to gene flow within the main stem Hudson River, although it is possible that tomcod in Newark Bay and its tributaries in the western estuary are largely isolated from those in the main stem of the Hudson River. Some support for the idea of reproductive isolation of tomcod in the western estuary comes from the different ratio of total PCDDs to PCDFs in tomcod from Newark Bay/Hackensack River compared with those in the main stem of the Hudson River (Courtenay et al. 1999). However, results from limited tagging studies have reported recaptures of tomcod in Newark Bay that were tagged in the Hudson River (Mattson M, personal communication), suggesting population movements between these sites. No genetic studies have empirically tested whether there are any spatial or temporal population subdivisions of tomcod within the estuary. It is known, however, that tomcod spawn over ≥ 50 miles of the main stem of the river extending from at least the Tappan Zee (RM 25) to Poughkeepsie, New York (RM 75) (Klauda et al. 1988). Spawning of tomcod almost certainly occurs in the Hackensack River, as evidenced by the seasonal abundance of young juveniles in early May (Wirgin II, Chambers RC, unpublished data). The likelihood of spawning in the Passaic River is unknown. Although tomcod eggs are demersal, yolk-sac larvae and older feeding larvae regularly ascend the water column and would be carried downstream. By late spring, juveniles are found throughout the lower estuary to waters adjacent to New York City. Considerable mixing of tomcod larvae from different spawning locales likely occurs during this process. It is likely that tomcod from throughout the Hudson River constitute a single panmictic population, and thus, the allelic variants underlying the resistant phenotypes are likely to be homogeneously distributed throughout the Hudson River population.

How geographically extensive is resistance in tomcod from the Hudson River compared with that reported in other species? Ownby et al. (2002) evaluated the extent of resistance to contaminated sediments in the F_1_ descendants of killifish collected from four sites on the Elizabeth River with varying levels of PAHs in their sediments and from a nearby reference locale, the York River, Virginia. They reported that the extent of resistance, as measured by the frequency of development of cardiovascular abnormalities [which is probably an AHR-mediated response (Dong et al. 2004; Teraoka et al. 2003)], differed significantly among sites on the Elizabeth River and between all Elizabeth River sites and the York River. This suggests that varying degrees of resistance had developed in killifish with differing exposure histories in the Elizabeth River. Nacci et al. (2002a) evaluated the degree of resistance in one and sometimes two generations of killifish whose parents were collected from 14 sites along the northeastern Atlantic coast of the United States from New Bedford Harbor to Tuckerton, New Jersey. The extent of resistance in offspring from these sites was compared with the surficial sediment concentration of total PCBs at each of the collection sites. They found that PCB-126 LC_50_ values (concentration lethal to 50%) of embryos and larvae differed significantly—in excess of 25,000-fold—among sites. Levels of resistance among collection sites as measured by tolerance to PCB-126 or survival were significantly correlated with sediment total PCBs (*r*^2^ = 0.968). Significant resistance was restricted to the offspring of fish collected within approximately 5 km of the PCB “hotspot” in upper New Bedford Harbor and from highly contaminated Newark Bay. Sediment concentrations of total PCBs at sites from which their descendents exhibited resistance exceeded 541 ng total PCBs/g dry sediment. Thus, the degree of resistance in killifish varied among sites and closely mirrored sediment concentrations of the agent thought to induce resistance.

We believe that the Hudson River hosts the most geographically extensive resistant population of any vertebrate reported in the literature. We found no variation in the degree of resistance to PCB-77 in fish collected over a 90-mile length of river despite the large variation in hepatic burdens of coplanar PCBs and PCDDs/PCDFs in juvenile tomcod from this area (Fernandez et al. 2004), including many of the same or nearby Hudson River sites from which tomcod were collected and treated for this study. Similarly aged juvenile tomcod that were the F_1_ descendants of parents collected from the western estuary in Newark Bay showed no evidence of gene induction by PCB-77, despite their being highly sensitive to induction by BaP. These laboratory-reared juveniles exhibited resistance despite their low tissue burdens of coplanar PCBs and PCDDs/PCDFs. These results suggest that resistance in Hudson River tomcod not only is prevalent throughout the estuary but also may be heritable to at least the F_1_ generation.

The difference in resistance patterns between tomcod and killifish almost certainly reflects their different propensity to move and their life history characteristics—limited mobility in killifish compared with estuary-wide movements in tomcod. It is likely that tomcod from the Hudson River estuary are reproductively isolated from the most proximal reproducing populations in Shinnecock Bay, New York, and in Long Island Sound, although this has not been empirically tested with population-genetic approaches. If this is so, after environmental remediation, the source of “sensitive” alleles will need to be variants from within the Hudson River population rather than migrants from elsewhere. We have already demonstrated that variation in *CYP1A* expression levels is high in environmentally exposed and chemically treated adult tomcod from the Hudson River (Courtenay et al. 1994), suggesting that its population contains individuals with differing *CYP1A* inducibility genotypes and therefore could serve as a source of sensitive fish to repopulate the river after its remediation.

In this study, tomcod from throughout the estuary exhibited significant CYP1A mRNA inducibility by BaP but not PCB-77. This result is consistent with dose–response studies previously conducted with adult tomcod collected from a single Hudson River locale, Garrison, New York, which showed significant inducibility of hepatic *CYP1A* by BaP and β-NF but not by a variety of coplanar PCBs or TCDD (Courtenay et al. 1999; Yuan et al. 2005). In contrast, killifish from the creosote-contaminated Elizabeth River were resistant to *CYP1A* induction by both PAHs (Meyer et al. 2002) and coplanar PCB-126 (Meyer and Di Giulio 2003), despite the absence of PCB contamination of that ecosystem. Killifish from PCB-contaminated New Bedford Harbor also exhibited resistance to both PAH and PCB induction of *CYP1A* (Nacci et al. 1999, 2002a), but less so for β-NF (a PAH) than for TCDD (Bello et al. 2001).

*CYP1A* transcription and most toxic responses to PCBs, PCDDs/PCDFs, and PAHs are believed to be mediated by the AHR pathway (Ma 2001). The persistence of halogenated AHs such as PCBs and PCDDs/PCDFs in fish tissues far exceeds that of PAHs. Hepatic PAHs are metabolized within hours (Varanasi and Stein 1991), whereas PCBs and PCDDs/PCDFs are often recalcitrant to metabolism and highly persistent, with half-lives in fish measured in weeks and months (Muir et al. 1992). As a result, the toxicities from exposures to halogenated AHs and nonhalogenated AHs are very different. Because of their reactivity, metabolites of PAHs are genotoxic by adducting to DNA and they are also acutely toxic, whereas the effects of HAHs are more chronic. Therefore, the selective pressure on tomcod populations from exposure to the two classes of AH toxicants may differ significantly. It has been demonstrated in marine fish species that metabolically refractory and persistent PCB-77 generates high levels of reactive oxygen species (ROS) within the active site of CYP1A and uncouples the catalytic cycle of CYP1A (Schlezinger et al. 1999). Because tomcod from the Hudson River bioaccumulate high levels of hepatic PCBs and PCDDs/PCDFs, it can be envisioned that down-regulation of CYP1A activities would result in lowered ROS and reduced cellular damage and therefore prove selectively advantageous. In this scenario, two distinct mechanisms of activation of *CYP1A* transcription would exist, one AHR dependent and a second that is AHR independent. In fact, some evidence of an AHR-independent pathway of PAH-induced toxicity has been demonstrated in rodent models (Bhat and Bresnick 1997; Dertinger et al. 2000).

Inhibition of *CYP1A* induction in its own right may have important consequences for the resistant population, but is it predictive of decreased sensitivity of Hudson River tomcod to toxicities at higher levels of biologic organization? In killifish from New Bedford Harbor (Nacci et al. 1999), Newark Bay (Prince and Cooper 1995a), and the Elizabeth River (Meyer et al. 2002; Ownby et al. 2002), reduced *CYP1A* inducibility co-occurs with decreased sensitivities to early-life-stage toxicities from exposure to AH compounds. In extensive dose–response studies with an environmentally relevant PCB mixture, TCDD (Wirgin and Chambers, in press), and individual PCB congeners (Wirgin II, Chambers RC, unpublished data), we observed significant differences between tomcod embryos from the Hudson River, Miramichi River, and Shinnecock Bay in sensitivities to morphologic malformations, hatching success, and behavioral deficits in emerging larvae. Early life stages of tomcod from the Hudson River were unaffected at these end points, whereas those from other populations were highly sensitive. Thus, reduced CYP1A mRNA inducibility in tomcod from the Hudson River is almost certainly predictive of reduced sensitivities at higher level toxic end points to coplanar PCBs and TCDD exposures. We have yet to compare sensitivities of the populations to PAH exposures.

Evolutionary costs of resistance to the Hudson River population of tomcod have yet to be identified, although the effects of coexposures to metals and BaP or PCB-77 are often different than those from either contaminant alone (Sorrentino et al. 2004, 2005). Offspring of killifish from the PAH-contaminated Elizabeth River were more sensitive to acute phototoxicity, ambient UV light, and low oxygen conditions than were those from reference locales and exhibited reduced growth and survivorship in the absence of contaminants (Meyer and Di Giulio 2003).

Regardless of the trade-off and the cost of tolerance to Hudson River tomcod, it is almost certain that resistant Hudson River tomcod are important sources of persistent PCBs and PCDDs/PCDFs at higher trophic levels within this ecosystem. The wide distribution and high abundance of juvenile tomcod throughout the tidal estuary in April through June, combined with its role as prey for many, diverse, and common fishes of the estuary, place tomcod at a critical node in the Hudson River food web. Not only do the spatial distributions of consumers of tomcod span the freshwater, estuarine, and marine portions of the estuary, but also these fishes differ in their residency within the estuary and hence their potential for translocating contaminants throughout and beyond the Hudson River ecosystem.

In summary, juvenile and adult tomcod from many Hudson River sites are highly contaminated with, yet resistant to, PCBs and PCDDs/PCDFs. The broad-scale resistance exhibited by tomcod from throughout the Hudson River estuary suggests a combination of high chronic exposure and a mobile pan-mictic population. This resistance is likely to have evolved relatively recently (the 1900s) and remains persistent despite cessation of most point source releases of PCBs and PCDDs/PCDFs. The full extent of community and ecosystem consequences of resistance in the Hudson River tomcod population is yet to be fully evaluated.

## Figures and Tables

**Table 1:** Hepatic burdens of coplanar PCBs, PCDDs, and PCDFs in juvenile Atlantic tomcod sampled from seven geographically distant sites in the Hudson River (HR) in 1998 and 2000, one site from the Miramichi River (MR) in 1998, and adult tomcod collected in Newark Bay (NB) in 1998.

	PCBs		PCDDs		PCDFs		Total TEQs	
Source	1998	2000	1998	2000	1998	2000	1998	2000
HR (RM)								
1	5	18	31	62	13	42	52	121
10	4		14		12		33	
17	13	7	35	239	31	175	85	420
37	15	11	16	17	27	11	68	38
43		10		5		4		19
80		10		14		13		37
91		9		7		12		28
MR		0.30		0.4		0.19		0.96
NB	15		671		50		741	

All concentrations are given in units of TCDD TEQs: for PCBs, sum of TCDD TEQs of four coplanar PCB congeners (International Union of Pure and Applied Chemistry numbers, 77, 81, 126, and 169); for PCDDs, sum of TCDD TEQs from PCDD congeners; for PCDFs, sum of TCDD TEQs from PCDF congeners; for total TEQs, total TCDD TEQs from PCB, PCDD, and PCDF congeners. These are a subset of the data expressed as wet and dry weight concentrations by Fernandez et al. (2004).
